# Enhanced Dy^3+^ white emission via energy transfer in spherical (Lu,Gd)_3_Al_5_O_12_ garnet phosphors

**DOI:** 10.1038/s41598-020-59232-8

**Published:** 2020-02-10

**Authors:** Jinkai Li, Wenzhi Wang, Bin Liu, Guangbin Duan, Zongming Liu

**Affiliations:** School of Materials Science and Engineering, University of Jinan, Jinan, Shandong 250022 China

**Keywords:** Materials science, Optics and photonics

## Abstract

The Dy^3+^ doped (Lu,Gd)_3_Al_5_O_12_ garnet phosphors with spherical morphology were obtained via homogeneous precipitation method, followed by calcination at 1100 °C. The particle morphology does not change significantly, but can be controlled by adjusting the urea content. The synthesis, structure, luminescent properties of precursor and resultant particles were analyzed by the combined technologies of XRD, FE-SEM, PLE/PL decay behavior. The (Lu_0.975_Dy_0.025_)AG phosphors display strong blue and yellow emission at ~481 nm (^4^F_9/2_ → ^6^H_15/2_ transition of Dy^3+^) and ~582 nm ^4^F_9/2_ → ^6^H_13/2_ transition of Dy^3+^), respectively. The phosphors have similar color coordinate and temperature of (~0.33, ~0.34), ~5517 K, respectively, which are closed to the white emission. The particle size and luminescent intensity decreased while the lifetime increased with the urea concentration increasing. The Gd^3+^ addition does not alter the shape/position of emission peaks, but enhance the blue and yellow emission of Dy^3+^ owing to the efficient Gd^3+^ → Dy^3+^ energy transfer. The [(Lu_1-x_Gd_x_)_0.975_Dy_0.025_]_3_Al_5_O_12_ phosphors are expected to be widely used in the lighting and display areas.

## Introduction

In the powder form, Ce^3+^ doped Ln_3_Al_5_O_12_ (LnAG) garnet phosphors, especially Ce^3+^ doped YAG (YAG:Ce), have become one of the most efficient yellow phosphors. YAG:Ce can be excited with blue light and thus be widely used in the rapidly expanding market of white light light emitting diodes (LEDs)^1–3^. Dy^3+^ doped YAG has been studied extensively by lots of researchers, and can be widely used in lighting and display areas^4^. While low color rendering and high correlated color temperature (CCT) are frequently deemed as drawbacks due to the lack of sufficient red spectral intensity. On the other side, the phosphor performance of YAG phosphors is needed to be further improved to fit the complicated lighting areas. In the bulk form, the YAG transparent ceramics can be applied to the solid state laser and scintillators, but the relative low density of ∼4.76 g/cm^3^ reduces stopping power and hinders its further development in scintillator areas^5^.

The spherical [(Lu_1-*x*_Gd_*x*_)_0.975_Dy_0.025_]AG phosphors developed in this work were chosen according to the following reasons: (1) the luminescent properties of phosphor were strongly dependent on the particle morphology and size^6^. The particle with uniform size and spherical morphology not only improves the resolution of fluorescent devices, but also facilitates compact fluorescent layer self-assembly easily. As a compact fluorescent layer, it can minimize the scattering of excitation light and present the best luminescence efficiency^7^; (2) compared with Dy^3+^-activated YAG and LuAG, the smaller electronegativity of Gd^3+^ (1.20) than that of Y^3+^ (1.22) and Lu^3+^ (1.27) may allow an easier charge transfer (CT), and thus may yield improved intensities of the CT/PL (photoluminescence) bands^8^. In addition, Gd^3+^ may sensitize the ^4^F_9/2_ → ^6^H_15/2_ and ^4^F_9/2_ → ^6^H_13/2_ emissions of Dy^3+^, through an efficient energy transfer from Gd^3+^ to Dy^3+^ ^9^, further improving the Dy^3+^ emission; (3) for scintillation applications, the material should have a high theoretical density to assure a high X-ray stopping power. The LuAG and GdAG maybe the best choice due to the heavier atom weight of Lu (175) and Gd (157) than Y (89), but the former price of raw material Lu_2_O_3_ is expensive and the GdAG is thermal instability^10–12^. In this regard, the (Lu,Gd)AG solid solution is the best desirable and could potentially be a new kind of scintillation material.

In the present work, (Lu,Gd)AG:Dy phosphors were calcined from their precursors synthesized via homogeneous coprecipitation with urea as the precipitant^13^. Phase evolution of the precursors upon calcination and photoluminescence behaviors of the oxide phosphors were studied in detail via the combined techniques of fourier transform infrared spectroscopy (FT-IR), X-ray diffractometry (XRD), field emission scanning electron microscopy (FE-SEM), photoluminescence excitation/photoluminescence (PLE/PL) spectroscopy, and fluorescence decay analysis. Luminescent properties of the (Lu,Gd)AG:Dy phosphors were successfully correlated to the particle size of the powder, and particularly the Gd^3+^ contents. In the following sections, we report the synthesis, characterization, and luminescent performance of the (Lu,Gd)AG:Dy garnet phosphors.

## Experiment Procedure

The starting chemicals used in this work are gadolinium oxide (Gd_2_O_3_, 99.99% pure, Huizhou Ruier Rare-Chem. Hi-Tech. Co. Ltd., Huizhou, China), lutetium oxide (Lu_2_O_3_, 99.99% pure, Huizhou Ruier), dysprosium oxide (Dy_2_O_3_, 99.99% pure, Huizhou Ruier), aluminum sulfate sulfate (NH_4_Al(SO_4_)_2_∙12H_2_O, 99.95% pure, Sinopharm Chemical Reagent Co., Ltd, Shanghai, China), aluminite nitrate (Al(NO_3_)_3_, 99.95% pure, Sinopharm Chemical Reagent Co., Ltd, Shanghai, China), urea (CO(NH_2_)_2_, >99% pure, Sinopharm Chemical Reagent Co., Ltd, Shanghai, China), and nitric acid (HNO_3_, excellent grade, Sinopharm Chemical Reagent Co., Ltd, Shanghai, China). All the reagents are used as received without further purification.

The rare earth nitrates Ln(NO_3_)_3_ (Ln = Gd, Lu, and Dy) were prepared by dissolving Ln_2_O_3_ oxides in proper amounts of hot nitric acid. The mother salts were prepared by mixing the rare earth nitrates Ln(NO_3_)_3_, (NH_4_Al(SO_4_)_2_∙12H_2_O and Al(NO_3_)_3_ according to [(Lu_1-*x*_Gd_*x*_)_0.975_Dy_0.025_]_3_Al_5_O_12_ formula. The urea as precipitant was blended to the mother solution in beaker and then dissolved to make total volume of 500 mL. The mixed solution was heated to 90 ± 1 °C within 1 h and reacted at 90 ± 1 °C for 2 h. After cooled to room temperature, the precipitate was collected via centrifugation, washed with distilled water, rinsed with ethanol, and dried at 80 °C for 12 h in air. The dried precursor was calcined in air at 1100 °C for 4 h to obtain oxides. In each case, the total mole concentration of Ln^3+^ and Al^3+^ ions were kept 0.03 and 0.05 mol/L, respectively. The mole ratio of NH_4_Al(SO_4_)_2_∙12H_2_O to Al(NO_3_)_3_ was kept 1:1. The Gd^3+^ content *x*, expressed as *x* = Gd/(Lu + Gd) atomic ratio (*x* = 0, 0.05, 0.10, 0.20, 0.30, 0.40, 0.50, and 0.60), was changed to reveal its effects on the properties of the precursor and the resultant [(Lu_1-*x*_Gd_*x*_)_0.975_Dy_0.025_]_3_Al_5_O_12_ garnet powders. The mole ratio *R*, expressed as *R* = urea/(Lu + Gd + Al) atomic ratio (R = 20, 40, 60, 80) was varied to investigate the urea content effect on the particle morphology and size.

The function group of the precursor was studied via fourier transform infrared spectroscopy (FT-IR, Model Nicolet 380, America). Phase identification was performed via X-ray diffractometry (XRD, Model D8-ADVANCE, BRUKER Co., Germany) using nickel-filtered Cu*K*α radiation and a scanning speed of 4° 2θ/min. Particle morphology was observed by field-emission scanning electron microscopy (FE-SEM, Model JSM-7001F, JEOL, Tokyo, Japan). Photoluminescence excitation (PLE) and photoluminescence (PL) spectra of the phosphors were analyzed using an FP-6500 fluorospectrophotometer (JASCO, Tokyo) equipped with a 60-mm-diamter intergating sphere (Model ISF-513, JASCO) and a 150-W Xe-lamp as the excitation source. The fluorescence decay kinetics of Dy^3+^ emission was measured at room temperature. The phosphor powder was excited with a selected wavelength and the intensity of the intended emission was recorded as a function of elapsed time after the excitation light was automatically cut-off by a shutter.

## Results and Discussion

**Figure 1:**
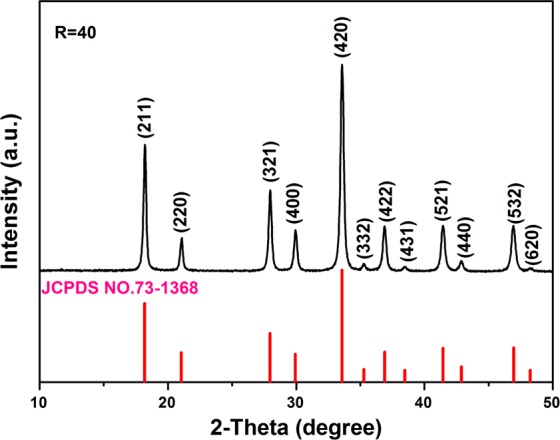
XRD pattern of the (Lu_0.975_Dy_0.025_)AG powders calcined at 1100 °C.

Figure 1 shows the XRD pattern of the (Lu_0.975_Dy_0.025_)_3_Al_5_O_12_ powders calcined at 1100 °C (*R* = 40). From which it can be seen that all the XRD diffraction peaks are well agreement with the standard PDF card of LuAG (cubic structure, JCPDS NO.73-1368). The Dy^3+^ addition does not alter the crystal structure of garnet phosphor.

**Figure 2:**
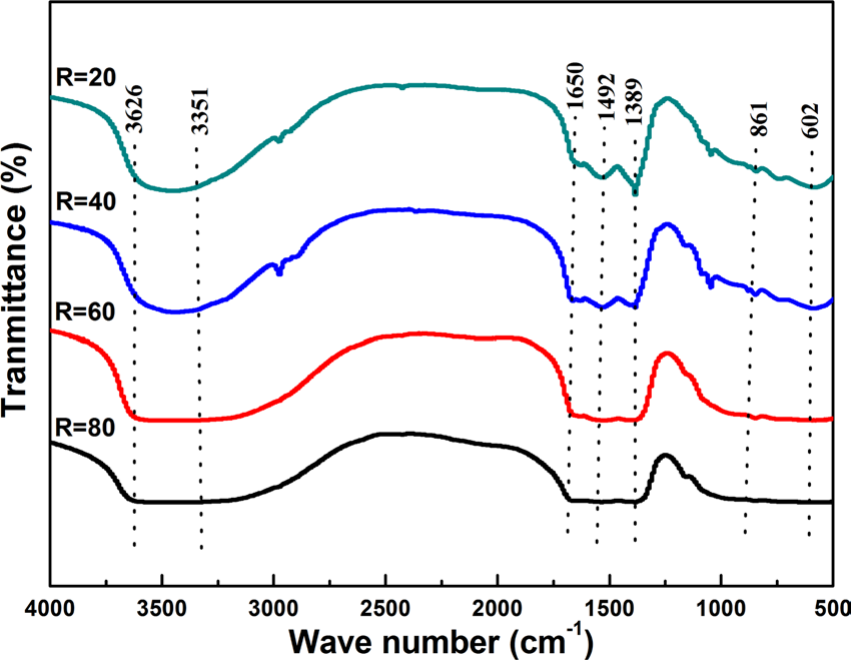
FT-IR analysis of (Lu_0.975_Dy_0.025_)AG precursor synthesized by different urea concentrations *R* = 20, 40, 60, and 80, respectively.

In order to investigate the function group composition in the precursor, the FT-IR analysis has been preformed and the results were displayed in Fig. 2. The strong and broad absorption bands centered at ~3351 cm^−1^ and the shallow shoulder near 1650 cm^−1^ are indicative of the water of hydration in the structure or surface adsorbed water^14–16^. The absorption band centered at ~3626 cm^−1^ provide evidence of the OH^−^ groups^14,15,17^. The appearance of absorptions peaking at ~1389 cm^−1^ and ~1492 cm^−1^ and ~861 cm^−1^ were ascribed to the presence of carbonate ions in the molecular structure^14–16^. The presence of M-O (M: metal ions) in the molecular structure was confirmed by the occurrence of absorption band centered at ~602 cm^−1^ ^15^. Based upon these FT-IR observations, the precursors obtained in work may be expressed with a general formula of (Lu_0.975_Dy_0.025_)Al_5_(OH)_*x*_(CO_3_)_*y*_·*n*H_2_O.

**Figure 3:**
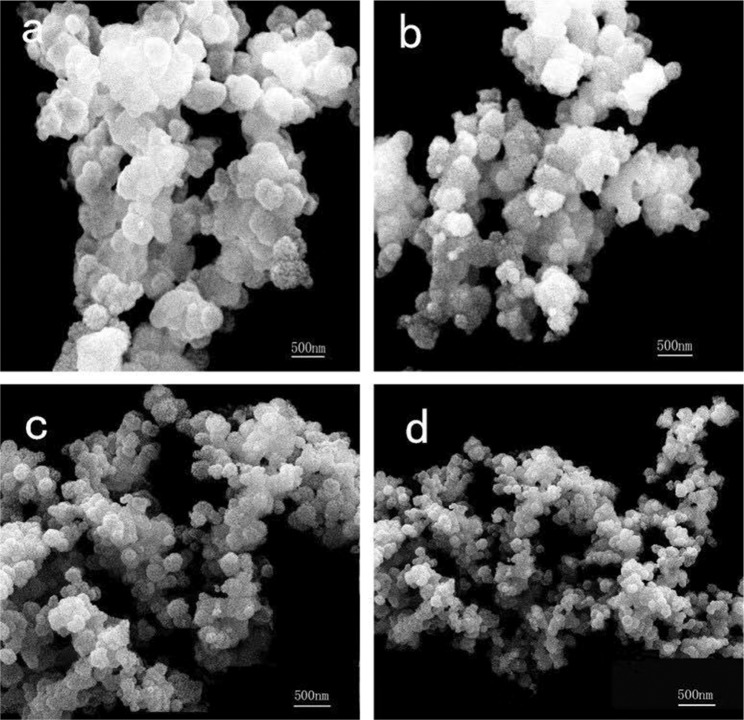
The FE-SEM micrograph of (Lu_0.975_Dy_0.025_)AG precursor synthesized with different urea concentrations (**a**) *R* = 20, (**b**) *R* = 40, (**c**) *R* = 60, (**d**) *R* = 80, respectively.

**Figure 4:**
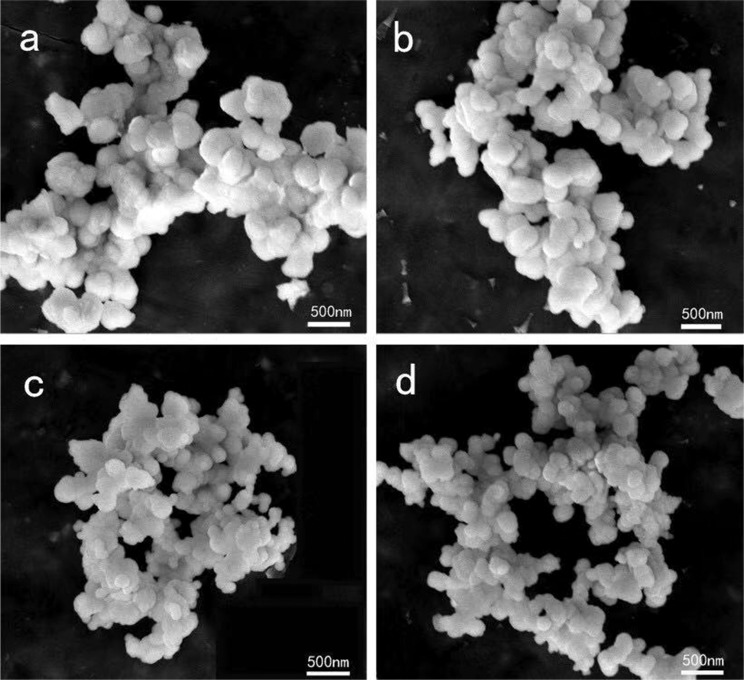
The FE-SEM micrograph of (Lu_0.975_Dy_0.025_)AG resultant products calcined at 1100 °C with different urea concentrations (**a**) *R* = 20, (**b**) *R* = 40, (**c**) *R* = 60, (**d**) *R* = 80, respectively.

Figure 3 displays the FE-SEM micrograph of (Lu_0.975_Dy_0.025_)AG precursor with different urea concentrations (*R* = 20, 40, 60, 80). The (Lu_0.975_Dy_0.025_)AG precursors exhibit a little agglomeration, and are composed of much fine particles due to the relatively strong adsorption capacity. Further observation is that the particle size decreased with the urea concentration increasing (Fig. 4a: *φ*_*a*_: ~450 nm, Fig. 4b: *φ*_*b*_: ~320 nm, Fig. 4c: *φ*_*c*_: ~230 nm and Fig. 4d: *φ*_*d*_: ~125 nm). This can be explained as follows: the higher urea concentration is, the more precipitator ion (OH^−^, CO_3_^2−^) produced by hydrolysis of urea (≥83 °C) which leads to the higher nucleation density. While the nucleation density is inversely proportional to the particle size, thus the decreased particle size was observed.

The micrograph of the resultant (Lu_0.975_Dy_0.025_)AG phosphors calcined at 1100 °C have also been investigated, and the results were shown in Fig. 4. From which it can be seen that the (Lu_0.975_Dy_0.025_)AG particle shows good dispersion even at high temperature of 1100 °C due to the good homogeneity of precursor. On the other side, the phosphors possess spherical morphology owing to the uniform release of OH^−^, CO_3_^2−^ in the hydrolysis process of urea^13^. When the mole ratio *R* equal to 20, 40, 60, and 80, the (Lu_0.975_Dy_0.025_)AG particle size was ∼390 nm, ∼260 nm, ∼200 nm, and ∼140 nm, respectively. The change trend of particle size as a function of urea concentration was similar to the precursor.

**Figure 5:**
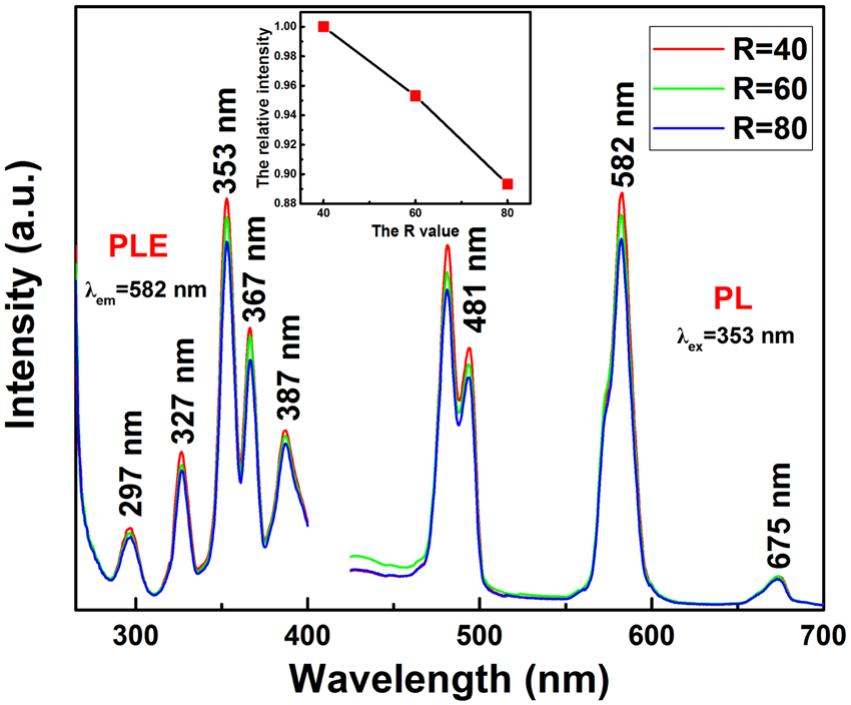
The PLE/PL spectra of (Lu_0.975_Dy_0.025_)AG phosphor calcined at 1100 °C as a function of urea concentration (*R*). The inset shows the relative PL intensity of 582 nm normalized to *R* = 40.

Figure 5 shows the PLE and PL properties of the (Lu_0.975_Dy_0.025_)AG phosphors calcined at 1100 °C. As seen in the figure, the PLE spectra possess similar shape for all the samples with different *R* values and consist five excitation peaks at ~297 nm, ~327 nm, ∼353 nm, ~367 nm, and ~387 nm ascribed to the matrix absorption, ^6^H_15/2_ → ^6^P_3/2_, ^6^H_15/2_ → ^4^I_11/2_ + ^4^M_15/2_ + ^6^P_7/2_, ^6^H_15/2_ → ^4^P_3/2_ + ^6^P_3/2_,_5/2_, and ^6^H_15/2_ → ^4^I_13/2_ + ^4^F_7/2_ + ^4^K_17/2_ + ^4^M_19/2,21/2_ of Dy^3+^ transitions^4,9,18^, respectively, with the ∼353 nm being dominant. Under the optimal excitation wavelength at 353 nm, the PL spectra displays strong blue emission (∼481 nm) and yellow emission (∼582 nm) due to the magnetic dipole ^4^F_9/2_ → ^6^H_15/2_ and electric dipole ^4^F_9/2_ → ^6^H_13/2_ transition of Dy^3+^, respectively^4,9,18^. Hardly perceptible at longer wavelength at ∼675 nm associated with the ^4^F_9/2_ → ^6^F_11/2_ transition of Dy^3+^ ^4,9,18^. Further observation was that the emission intensity decreased with the *R* values increasing. This reason can be explained as follows: the higher the urea concentration, the smaller the particle size as seen from the inset of Fig. 4. While the smaller particle size possess the bigger specific surface area, and the more defects formation on the particle surface leading to the weakened emission intensity. In addition, the agglomeration of small particles is also one of the reasons for the weak fluorescence intensity.

**Figure 6:**
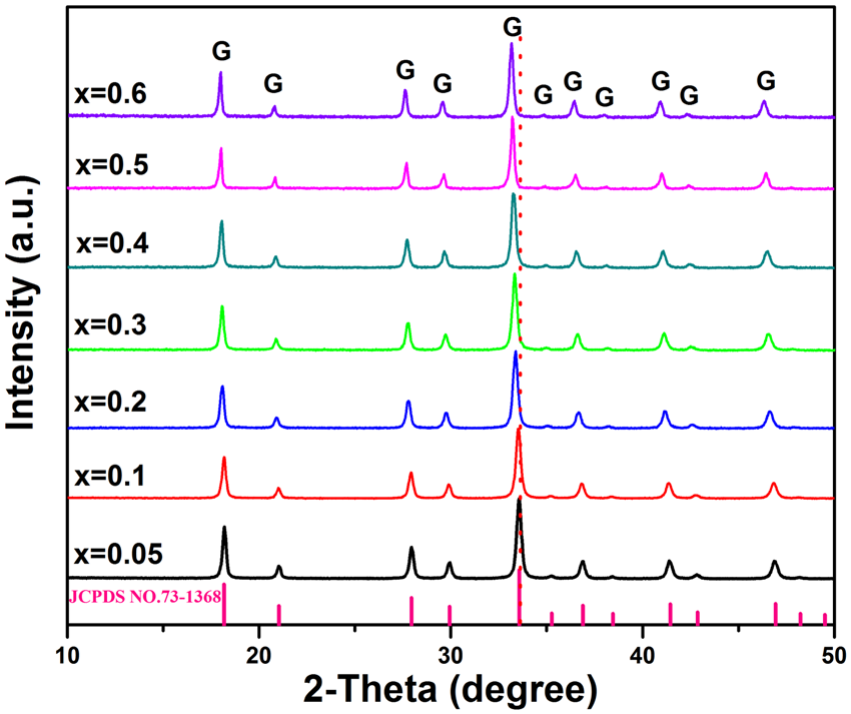
XRD patterns of the [(Lu_1-*x*_Gd_*x*_)_0.975_Dy_0.025_]AG powders calcined at 1100 °C as function of Gd^3+^ content (the *x* value marked in the figure).

Figure 6 shows the XRD patterns of [(Lu_1-*x*_Gd_*x*_)_0.975_Dy_0.025_]AG particles with the increased Gd^3+^ content up to *x* = 0.6. The Gd^3+^ addition does not alter the crystal structure of the resultant phosphor particle, and all the XRD patterns confirm with the XRD standard card of LuAG (No: 73–1368) with the cubic structure. Further observation was that the XRD peaks shift towards the lower angle using the (420) peak as an example which lead to the lattice expansion due to the larger ion radius of Gd^3+^ than Lu^3+^ (for 8-fold coordination, Gd^3+^ and Lu^3+^ have their respective ionic radii of 0.1053, and 0.0977 nm)^19^. It should be noticed that the maximum doped content of Gd^3+^ is 60% (*x* = 0.6). This is mainly because the following two reasons: (1) the thermal stability of LnAG strongly depends the ion radius of Ln^3+^. The Gd^3+^ doping would increase the average ion radius of rare earth, when the content of Gd^3+^ is over 60% will result in the decomposition of [(Lu_1-*x*_Gd_*x*_)_0.975_Dy_0.025_]AG to [(Lu_1-*x*_Gd_*x*_)_0.975_Dy_0.025_]AlO_3_ and Al_2_O_3_ compounds^20^; (2) the spherical morphology could not be kept if the Gd^3+^ content exceeds 60% (*x* > 0.6) in our present work.

**Figure 7:**
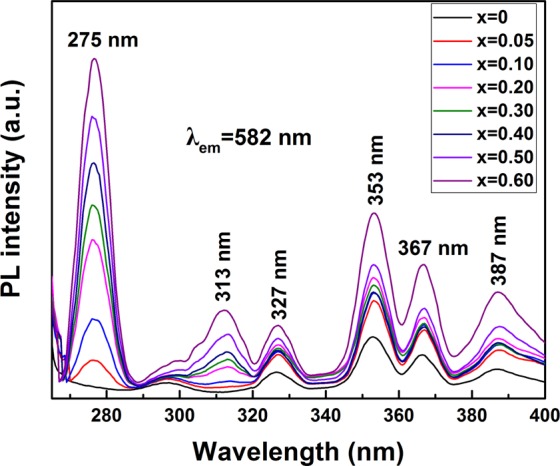
A comparison of the PLE behaviors of the [(Lu_1-*x*_Gd_*x*_)_0.975_Dy_0.025_]AG (*x* = 0–0.6) phosphors calcined at 1100 °C. The PLE spectra were obtained by monitoring the 582 nm emission.

Keeping Dy^3+^ at the optimal content of 2.5 at%^9^, the effects of Gd^3+^ concentration on PLE properties of the phosphors are studied in Fig. 7. The Gd^3+^-containing samples clearly exhibit a strong PLE band at ~275 nm and a weak one at ~313 nm ascribed to the ^8^S_7/2_ → ^6^I_J_ and ^8^S_7/2_ → ^6^P_J_ intra *f-f* transitions of Gd^3+^^21,22^, suggesting that the energy transfer from Gd^3+^ to Dy^3+^ takes place in these samples. The other excitation bands at ~327 nm, ∼353 nm, ~367 nm and ~387 nm have similar PLE behavior with the (Lu_0.975_Dy_0.025_)AG sample. Further observation was that replacing Lu^3+^ with Gd^3+^ up to 60 at% does not alter appreciably peak positions but tends to strengthen the intensities of both the Gd^3+^ and Dy^3+^ excitation bands, owing to the lower electronegativity of Gd^3+^ (1.20) than Lu^3+^ (1.27).

**Figure 8:**
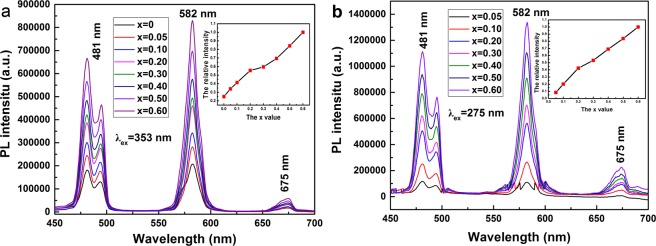
PL behaviors of the [(Lu_1-*x*_Gd_*x*_)_0.975_Dy_0.025_]AG (*x* = 0–0.6) calcined at 1100 °C. The PL spectra in panel (a) were obtained under 353 nm excitation. The PL spectra in panel (b) were all obtained under 275 nm excitations. Insets are the relative intensity of the 582 nm emission as a function of the Gd content, where the relative intensities were obtained by normalizing the observed 582 nm PL intensities to that of the [(Lu_0.4_Gd_0.6_)_0.975_ Dy_0.025_]AG sample.

The PL spectra of [(Lu_1-*x*_Gd_*x*_)_0.975_Dy_0.025_]AG (*x* = 0–0.6) phosphors with different Gd^3+^ addition under 353 nm and 275 nm excitation were displayed in Fig. 8. Whether using 353 nm or 275 nm excitation, all the samples in our work exhibit the similar PL bands at ∼481 nm, ∼582 nm, and ∼675 nm ascribed to the ^4^F_9/2_ → ^6^H_15/2_, ^4^F_9/2_ → ^6^H_13/2_ and ^4^F_9/2_ → ^6^H_11/2_, respectively^4,9,18^. Owing to the Gd^3+^ → Dy^3+^ energy transfer, the Gd-containing phosphors possess significantly stronger Dy^3+^ emissions under 275 nm than 353 nm excitation demonstrated by the comparison between parts (a) and (b) of the PL spectra.

The 582 nm emission under 275 nm excitation has an intensity roughly ~0.392, ~0.774, ~1.221 ~1.422, ~1.581, ~1.584 and ~1.588 times that under 353 nm excitation for *x* = 0.05, 0.1, 0.2, 0.3, 0.4, 0.5, and 0.6, respectively. Almost linearly increased emission intensities were observed with more Gd^3+^ incorporation, dominantly owing to the decreased electronegativity of the (Lu_1-*x*_Gd_*x*_)^3+^ pair (under both 275 and 353 nm excitation). The electronegativity of the (Lu_1-*x*_Gd_*x*_)^3+^ pair was determined to be ~1.267, ~1.263, ~1.256, ~1.249, ~1.242, ~1.235 and ~1.228 for *x* = 0.05, 0.1, 0.2, 0.3, 0.4, 0.5, and 0.6, respectively. It is also delighted for us to see that, with the efficient Gd^3+^ → Dy^3+^ energy transfer, the [(Lu_1-*x*_Gd_*x*_)_0.975_Dy_0.025_]AG phosphors under 275 nm excitation have 582 nm emission intensities ~0.53, ~1.28, ~2.72, ~3.39, ~4.39, ~5.33, and ~6.36 times of (Lu_0.975_Dy_0.025_)AG emission for *x* = 0.05, 0.1, 0.2, 0.3, 0.4, 0.5, and 0.6, respectively (Fig. 8b). Even under the same excitation at 353 nm (Fig. 8a), the [(Lu_1-*x*_Gd_*x*_)_0.975_Dy_0.025_]AG phosphors show 582 nm emission intensities ~1.36 (*x* = 0.05), ~1.65 (*x* = 0.1), ~2.22 (*x* = 0.2), ~2.38 (*x* = 0.3), ~2.78 (*x* = 0.4), ~3.37 (*x* = 0.5), and ~4.00 (*x* = 0.6) times of (Lu_0.975_Dy_0.025_)AG emission. The PL results shown in Fig. 8 suggest that the Gd content should be maximized to achieve better Dy^3+^ emission as along as the GdAG lattice and spherical morphology can be maintained.

**Figure 9:**
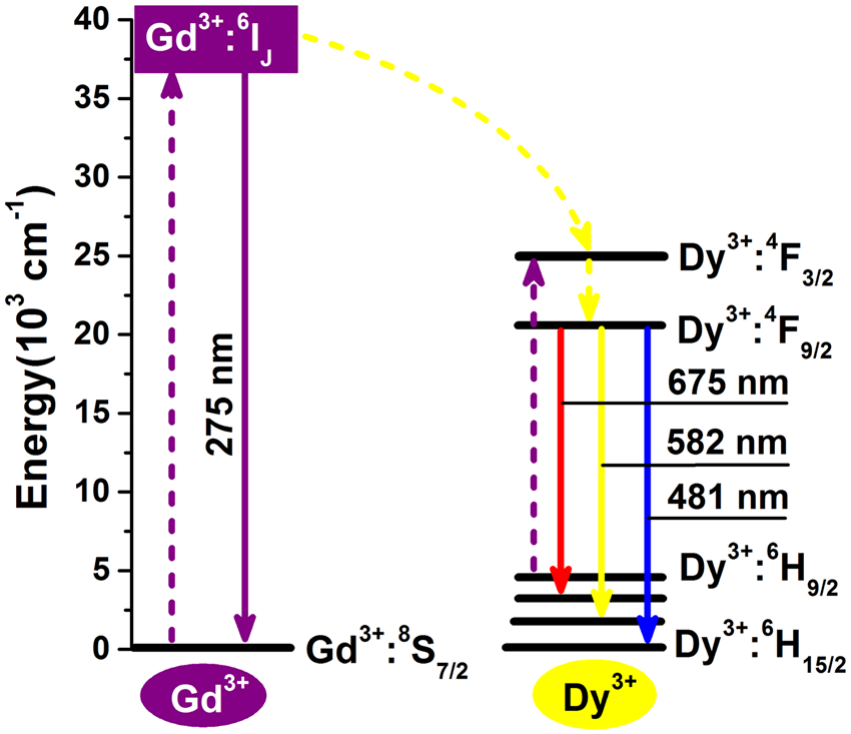
Illustration of the energy-transfer processes for the [(Lu_1-*x*_Gd_*x*_)_0.975_Dy_0.025_]AG phosphors.

The above analysis indicate that the Gd^3+^ → Dy^3+^ energy transfer may exist in the [(Lu_1-*x*_Gd_*x*_)_0.975_Dy_0.025_]AG phosphors which was shown in Fig. 9. By monitoring the 275 nm excitation wavelength, the electrons of the ^8^S_7/2_ ground state though absorbing the energy can transmit to the ^6^I_J_ excited state of Gd^3+^, and at the same time excites the ^6^H_15/2_ electrons of Dy^3+^ to the ^4^F_3/2_ states. The energy transfer of the Gd^3+^ → Dy^3+^ may happen owing to that the ^4^F_3/2_ (Dy^3+^) state in the energy diagram lies lower than the ^6^I_J_ state of Gd^3+^. Then the electrons of Dy^3+^ relaxed from ^4^F_3/2_ to ^4^F_9/2_.

**Figure 10:**
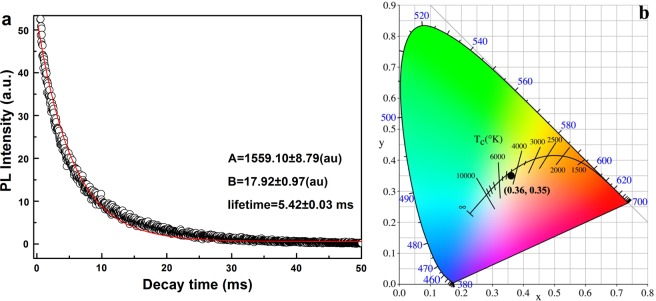
(**a**) Fluorescence decay kinetics for the 582 nm emission (λ_ex_ = 275 nm) of [(Lu_0.4_Gd_0.6_)_0.975_Dy_0.025_]AG calcined at 1100 °C for 4 h. (**b**) CIE chromaticity coordinate and color temperature of the [(Lu_1-x_Gd_x_)_0.975_Dy_0.025_]AG phosphors calcined at 1100 °C as function of Gd^3+^ content *x* (*x* = 0–0.6).

In order to investigate the luminescent lifetime of the phosphor synthesized in this work, the decay behavior analysis has been performed using the [(Lu_0.4_Gd_0.6_)_0.975_Dy_0.025_]AG (*x* = 0.6) sample calcined at 1100 °C as an example (Fig. 10a). The decay curve can be fitted to a single exponential according to the equation:

1 $$I=A\exp (-t/{\tau }_{R})+B$$

where *τ*_*R*_ is the fluorescence lifetime, *t* the delay time, *I* the relative intensity, and *A* and *B* are constants. The exponential fitting yields *τ*_*R*_ = 5.42 ± 0.03 ms, A = 1559.10 ± 8.79, B = 17.92 ± 0.97, respectively. The phosphors were found to have similar fluorescence lifetimes, irrespective of the excitation wavelength (275 nm or 353 nm) and Gd^3+^ content. Figure 10b displays the CIE chromaticity coordinates and color temperature of the [(Lu_1-x_Gd_x_)_0.975_Dy_0.025_]AG phosphors calcined at 1100 °C. It can be seen that the phosphor with different Gd^3+^ content have similar CIE chromaticity coordinates of (~0.36, ~0.35), close to the point of (0.33, 0.33) for an ideal white-color in the chromaticity diagram and has a color temperature of ~5517 K.

The color purity is an important property of the phosphor chromaticity property, and the color purity can be calculated via the following formula:

2 $$Colorpurity=\frac{\sqrt{{(x-{x}_{i})}^{2}+{({x}_{d}-{x}_{i})}^{2}}}{\sqrt{{(y-{y}_{i})}^{2}+{({y}_{d}-{y}_{i})}^{2}}}\times 100 \% $$

where (*x*, *y*) is the color coordinate of the light source, (*x*_*i*_, *y*_*i*_) is the CIE of an equal-energy illuminant with a value of (0.3333,0.3333), and (*x*_*d*_, *y*_*d*_) is the chromaticity coordinate corresponding to the dominant wavelength of the light source. We can obtain the (*x*_*d*_, *y*_*d*_) chromaticity coordinate of [(Lu_1-*x*_Gd_*x*_)_0.975_Dy_0.025_]AG by referring to the literature. By substituting the coordinates of (x, y), (*x*_*i*_, *y*_*i*_), and (*x*_*d*_, *y*_*d*_) in Eq. (2), the color purities of [(Lu_1-*x*_Gd_*x*_)_0.975_Dy_0.025_]AG, are determined to be ~92.3% indicating the vivid white color emission.

## Inclusions

The [(Lu_1-x_Gd_x_)_0.975_Dy_0.025_]AG (*x* = 0–0.6) garnet phosphors in the present work with monodisperse spherical morphology were obtained via homogeneous precipitation method at 1100 °C. The resultant phosphor were studied by the combined technologies of FT-IR, XRD, FE-SEM, PLE/PL, luminescent decay analysis, etc, and the results were summarized as follows:

1. The chemical formula of precursor can be expressed as (Lu_0.975_Dy_0.025_)Al_5_(OH)_*x*_(CO_3_)_*y*_·*n*H_2_O in principle. The phosphor particle size can be governed by changing the urea content, and decrease with the urea content increasing;
2. Significantly stronger Dy^3+^ emission can be achieved via indirectly exciting the Gd^3+^ at ∼275 nm (the ^8^S_7/2_ → ^6^I_J_ transition of Gd^3+^) rather than directly the Dy^3+^ at ∼353 nm (the ^6^H_15/2_ → ^4^I_11/2_ + ^4^M_15/2_ + ^6^P_7/2_ transition of Dy^3+^) for Gd^3+^-containing samples which indirectly proved the Gd^3+^ → Dy^3+^ energy transfer. The phosphors display strong blue (~481 nm, the ^4^F_9/2_ → ^6^H_15/2_ transition of Dy^3+^) and yellow (~582 nm, the ^4^F_9/2_ → ^6^H_13/2_ transition of Dy^3+^) emissions, with CIE chromaticity coordinates and color temperature of (~0.36, ~0.35) and ~5517 K, respectively;
3. Owing to the efficient Gd^3+^ → Dy^3+^ energy transfer, the luminescent properties of Gd^3+^-containing samples were much better than the Dy^3+^ doped pure LuAG sample. The best luminescent [(Lu_0.4_Gd_0.6_)_0.975_Dy_0.025_]AG (*x* = 0.6) phosphor has an intensity of the 582-nm emission (λ_ex_ = 275 nm) ~6.36 time of those of the (Lu_0.975_Dy_0.025_)AG phosphors (λ_ex_ = 353 nm), respectively;
4. There is a close relationship between the luminescent intensity and the particle size and Gd^3+^ content (*x* value), and the luminescent intensity increased with the particle size and the Gd^3+^ content increasing;
5. The lifetime of phosphors was determined to be 5.42 ± 0.03 ms, and the excitation wavelength (275 nm or 353 nm) and Gd^3+^ content have little effect on the phosphor lifetime.
